# Consumption of Ultra-Processed Food and Drink Products in a Greek Christian Orthodox Church Fasting Population

**DOI:** 10.3390/nu15234907

**Published:** 2023-11-24

**Authors:** Anna Kokkinopoulou, Niki Katsiki, Ioannis Pagkalos, Nikolaos E. Rodopaios, Alexandra-Aikaterini Koulouri, Eleni Vasara, Sousana K. Papadopoulou, Petros Skepastianos, Maria Hassapidou, Anthony G. Kafatos

**Affiliations:** 1 Department of Preventive Medicine and Nutrition Unit, School of Medicine, University of Crete, 70013 Crete, Greecekafatos@med.uoc.gr (A.G.K.); 2 Department of Nutritional Sciences and Dietetics, International Hellenic University, 57400 Thessaloniki, Greeceipagkalos@ihu.gr (I.P.); sousana@the.ihu.gr (S.K.P.); 3 School of Medicine, European University Cyprus, 2404 Nicosia, Cyprus; 4 Laboratory of Animal Physiology, Department of Zoology, School of Biology, Aristotle University of Thessaloniki, 54124 Thessaloniki, Greece; 5 Department of Medical Laboratory Studies, International Hellenic University, 57400 Thessaloniki, Greece

**Keywords:** ultra-processed foods, processed foods, unprocessed or minimally processed foods, Christian Orthodox church fasting, NOVA, metabolic syndrome

## Abstract

The positive effects of the Mediterranean diet on healthy living are widely known, while the health effects of religious fasting have received increased attention during the last decade. However, no study has focused on the consumption of ultra-processed foods (UPFs) in such populations. Therefore, our aim was to investigate UPF intake and its association with metabolic syndrome (MetS) in a Christian Orthodox church (COC) religious fasting population in Greece. In this cross-sectional study, 400 individuals who follow the Mediterranean diet were included, stratified as COC fasters and non-fasters. Dietary intake data were collected via three 24 h diet recalls and a monthly food frequency questionnaire (FFQ). The NOVA food classification system was used to identify the level of processing and categorize all food items. Fasters consumed significantly less chicken, turkey, and beef and significantly more seafood, fish, snails, soy products, and fresh fruits, in terms of unprocessed or minimally processed foods, as well as significantly more fried potatoes and olives in terms of processed foods when compared with non-fasters. Regarding UPFs, a significantly lower intake of pork sausages, ketchup, and mustard as well as a significantly higher consumption of margarine and tarama dip were recorded in fasters compared with non-fasters. Fasters with MetS more frequently consumed UPFs (such as cheese pastries, biscuits, and cakes) than fasters without MetS (*p* < 0.05 for all comparisons). Similarly, non-fasters with MetS had an increased intake of UPFs (such as Cypriot bread and Coco Pops breakfast cereals) than non-fasters without MetS. Future research should focus on UPF consumption and its associations with clinical outcomes in such populations, thus providing further data for the potential health effects of COC fasting.

## 1. Introduction

The Mediterranean diet is one of the most studied dietary patterns worldwide, and it has been associated with healthy living, prevention of non-communicable diseases, and longevity [1,2,3,4,5,6]. The fundamentals of the Mediterranean diet were first studied by Ancel Keys in the Seven Countries Study (SCS) in 1960, when the Cretan diet was firstly documented [7]. Notably, Christian Orthodox church (COC) fasting was the main characteristic of the Cretan diet in the SCS [7]. The COC fasting regime is followed both by believers and as a part of the tradition in Greece for 180–200 days annually, i.e., during Christmas (40 days), Easter (48 days), Assumption (14 days), and Holy Apostoles (0–30 days depending on Easter) fasting periods; every Wednesday and Friday; and during three more daily feasts (5 January, 29 August, 14 September) [7]. The COC fasting recommendations include avoidance of red and white meat, eggs, and dairy products as well as olive oil and wine during some days; fish and seafood is allowed. Therefore, this unique dietary pattern changes from a mixed Mediterranean diet to a vegetarian and pescatarian diet [7].

Ultra-processed foods (UPFs) are a category of food products that have undergone extensive industrial processing and, typically, contain a multitude of additives, thus often resulting in a product that is highly palatable, convenient, and has a long shelf life [8]. These foods are commonly made from cheap ingredients and sometimes are heavily marketed by the food industry. Furthermore, they usually contain high amounts of salt, sugar, unhealthy fats, and various artificial ingredients and are high in energy density [5]. It follows that UPFs have been associated with negative health effects, especially when consumed in excess. In this context, regular consumption of UPFs has been linked to several cardiometabolic disorders such as obesity, type 2 diabetes, heart diseases, high blood pressure (BP), and metabolic syndrome (MetS) [8,9,10,11]. In contrast, a diet based on whole, unprocessed, and/or minimally processed foods, including fruits, vegetables, whole grains, legumes, and healthy fats, such as the Mediterranean diet, the COC dietary pattern, and/or a plant-based diet, is generally considered more beneficial for overall health and well-being [12,13,14].

Previous studies reported a significant positive association between UPF intake and MetS prevalence [15], including a recent meta-analysis, of 9 studies with 23,500 participants, that found a 25% increased relative risk of developing MetS between the highest vs. lowest UPF consumption category in adults [16]. Of note, the joint criteria for MetS diagnosis include: (i) increased waist circumference (according to ethnicity-specific cut-off points), (ii) elevated fasting blood glucose, (iii) reduced high-density lipoprotein (HDL) cholesterol, (iv) increased fasting triglycerides, and (v) elevated BP [17]. Several factors might contribute to the observed link between UPF consumption and MetS incidence. For example, UPFs are often energy-dense but lack essential vitamins, minerals, and other beneficial nutrients [18]. Consuming these foods regularly can lead to overeating and weight gain, thus increasing the risk of obesity, which is a major component of MetS [19]. As mentioned above, UPFs also contain salt, sugar, unhealthy trans and saturated fats (which can raise BP), glucose, and lipids (total cholesterol, triglycerides, and low density lipoprotein (LDL) cholesterol), thus predisposing consumers to the development of MetS and other cardiometabolic diseases [20]. Furthermore, the lack or low percentage of dietary fibers in UPFs can affect digestion and satiety, thus negatively affecting the gut microbiome [21]. Overall, UPFs are frequently a part of the “Western diet” that has been linked to an increased risk of MetS [22,23].

To the best of our knowledge, no study to date has investigated the consumption of UPFs in a COC religious fasting population. Therefore, in the present study, we aimed to classify foods, according to their processing, using the NOVA classification [18,24], as well as to examine the associations between UPF intake and MetS presence in a Greek COC fasting population.

## 2. Materials and Methods

### 2.1. Study Population

In this cross-sectional study, 400 individuals were recruited on a voluntary basis through a call in public universities, monasteries, and churches in Thessaloniki, the second-largest city of Greece. The aims and the protocol of the study were clearly described to all individuals that expressed interest in participating, while any questions were answered by researchers. Overall, 400 individuals (mean age 42.6 ± 17.0 years, 257 women) were finally included in the present analysis among the initial 454 individuals who gave consent to participate. Due to inclusion criteria, individuals who were not able to provide informed consent and did not participate in all anthropometric and biochemical data collection were excluded from the study. Individuals who fasted regularly since their childhood, or for at least the last twelve consecutive years, formed the COC fasting group (*n* = 200, 131 women), whereas those who did not fast and/or follow any other restrictive dietary pattern, such as avoidance of wheat and/or lactose, formed the non-fasting group (*n* = 200, 126 women). More details about the inclusion/exclusion criteria and the study population have been described previously [25,26]. The protocol was approved by the Bioethics Committee of the Alexander Technological Educational Institute of Thessaloniki (ΔΦ 31-5/5679, date 17 December 2013).

### 2.2. Main Outcome Measurements

A validated questionnaire based on the nutrition care process plan model [27] was used for anthropometric, biochemical, clinical, dietary, and environmental data collection [28]. A trained nutritionist performed all anthropometric measurements and supervised the completion of the questionnaires. Prior to the scheduled appointment, all participants were asked to fast for at least 10 h and to abstain from physical activity for 24 h. On the day of the appointment, all participants filled a questionnaire with yes/no and open- and closed-ended questions to gather information about their lifestyle habits and socioeconomic status. For dietary intake data collection, three 24 h diet recalls [29], one food frequency questionnaire (FFQ) [30], and a nutritional behavior questionnaire were used [28]. Regarding the three 24 h diet recalls, one was collected on the day of the appointment, and the others were carried out via telephone. With regard to the FFQ, for each food item, the participants chose one of the following options: (i) never/rarely, (ii) one–three times/month, (iii) one–two times/week, (iv) three–six times/week, (v) one time/day, and (vi) >two times/day. Food replicas and household measures were used for the accuracy of portion sizes, whereas food records were analyzed by the Food Processor v11.7 nutrition analysis software (ESHA, Salem, OR, USA). Apart from the foods already found in the Food Processor software, Greek food recipes from the Greek food composition tables [31] were added in the software’s database. All diet recalls were entered in the software and mean values for nutrient and energy intake were extracted for each participant. All outcome measurements were collected by 400 participants, apart from the FFQ and the three 24 h diet recalls that were fully answered by 350 participants (174 fasters and 176 non-fasters).

BP was measured with an electronic BP monitor (Omron, Hoffman Estates, IL, USA), and waist circumference was measured with an SECA body girth tape (SECA 201, Hamburg, Germany). Fasting blood samples were also collected; more details on biochemical analyses and anthropometric measurements have been previously published [25].

### 2.3. UPFs and NOVA Classification

The NOVA food classification system is used by researchers to classify all food and drink products in four categories, based on the extent of their process before consumption [18,24,32]. The NOVA group 1 includes “unprocessed or minimally processed foods”, i.e., foods that come directly from nature and have no added ingredients, such as fresh fruits and meat. The NOVA group 2 contains the “processed culinary ingredients”, such as salt, sugar, olive oil and starch, that are derived from NOVA group 1 foods. The NOVA group 3 includes the “processed foods”, i.e., foods and drinks that are derived from combining the NOVA group 1 and 2 foods, such as homemade cooked foods, canned food products, and cured meats among others. Lastly, the NOVA group 4 contains the “UPFs”, i.e., the ready-to-eat food and drink products that have little to none NOVA group 1 ingredients, such as sweet packaged snacks and fizzy drinks [18,24,32].

### 2.4. Statistical Methods

Statistical analysis was performed with the use of the SPSS version 21 software (SPSS, Chicago, IL, USA). A two-sided *p*-value of 0.05 was set for statistical significance. The chi-squared test, one-way analysis of variance (ANOVA), and logistic regression analyses were performed to investigate any differences in UPF consumption between fasters and non-fasters, for gender analysis, and to evaluate any associations between UPF intake and MetS incidence.

## 3. Results

Overall, 200 fasters (131 women) and 200 non-fasters (126 women) participated in our cross-sectional study, with no differences between the age group in terms of sex representation. Furthermore, no differences were observed in both the education (*p* = 0.17) and the marital status (*p* = 0.67) between fasters and non-fasters. Significantly more non-fasters were smokers (*n* = 58 vs. 11 fasters, *p* < 0.001), they were watching TV significantly longer (2.2 ± 1.7 vs. 1.1 ± 1.3 h/day, *p* < 0.001), they were spending significantly more time with screens (PC/mobiles) (2.9 ± 2.9 vs. 2.3 ± 2.9 h/day, *p* = 0.043), and they were napping significantly more during the day (1.2 ± 1.4 vs. 0.8 ± 0.9 h/day, *p* = 0.008). Results regarding the demographics and lifestyle characteristics of the study sample can be found in Table 1.

The FFQ was answered by 350 individuals (174 fasters) and originally included 153 food and drink items. Only those items consumed by ≥5% of our study population were analyzed according to the NOVA classification system, as shown in Table 2.

With regard to NOVA group 1 items, i.e., the unprocessed or minimally processed foods, fasters consumed significantly less meat products and more seafood and fish products compared with non-fasters. In more detail, non-fasters consumed significantly more chicken at least once per week (*n* = 53 vs. *n* = 36 fasters, *p* = 0.001), turkey (*n* = 10 vs. *n* = 3 fasters, *p* = 0.003), and beef (*n* = 67 vs. *n* = 55 fasters, *p* = 0.007). On the other hand, fasters consumed significantly more seafood at least once per week (*n* = 115 vs. *n* = 101 non-fasters, *p* = 0.008), more fresh fish (*n* = 163 vs. *n* = 153 non-fasters, *p* = 0.022), and more snails (*n* = 9 vs. *n* = 2 non-fasters, *p* = 0.026). Furthermore, fasters consumed significantly more soya products at least once per week (*n* = 28 vs. 13 non-fasters, *p* = 0.001) and more fresh fruits at least once per day (*n* = 120 vs. *n* = 104 non-fasters, *p* = 0.022). These results are shown in Supplementary Table S2.

No significant differences were observed in relation to the consumption of the NOVA group 2 items (i.e., “processed culinary ingredients”) between fasters and non-fasters. Results regarding these food items can be seen in Supplementary Table S3.

Regarding the intake of NOVA group 3 items (i.e., “processed foods”), significant differences were found only for fried potatoes and olives. Briefly, significantly more non-fasters (*n* = 74) consumed fried potatoes at least once per week compared with fasters (*n* = 57, *p* = 0.001), whereas more fasters (*n* = 38) consumed olives every day vs. non-fasters (*n* = 20, *p* = 0.006). Results for the NOVA group 3 are shown in Supplementary Table S4.

With regard to the NOVA group 4 foods, i.e., UPFs, significantly more fasters rarely consumed pork sausages (108 fasters vs. 88 non-fasters, *p* = 0.003), ketchup (119 fasters vs. 9 non-fasters, *p* = 0.002), and mustard (79 fasters vs. 63 non-fasters, *p* = 0.014). Furthermore, more fasters consumed margarine one to two times per day compared with non-fasters (14 fasters vs. 12 non-fasters, *p* = 0.024) and more fasters consumed tarama dip more than once a week (36 fasters vs. 21 non-fasters, *p* = 0.041). No other significant differences were observed, and results are shown in Supplementary Table S5.

When analysis was carried out based on gender, a few significant differences were found in the consumption of specific foods. In more detail, regarding consumption of NOVA group 1 “unprocessed or minimally processed foods”, significantly more women were drinking herbal tea at least three or more times per week (*n* = 42 vs. *n* = 16 men, *p* = 0.009), and more women consumed fresh vegetables at least once per day (*n* = 140 vs. *n* = 61 men, *p* = 0.031). In terms of NOVA group 2 “processed culinary products”, significantly more women consumed honey at least once per day and/or more (*n* = 54 vs. *n* = 19, respectively, *p* = 0.042). In regard to the consumption of NOVA group 3 “processed foods”, it was shown that women consumed at least one portion daily of whole fat cheese significantly more often (*n* = 81 vs. *n* = 32 men, *p* = 0.027). Lastly, in terms of NOVA group 4 “UPFs” consumption, no significant difference was found.

According to gender analysis in fasters, the only significant difference found in relation to food intake was in the NOVA group 4 “UPFs”, with men fasters consuming tarama dip from three to six times per week significantly more often (24.6% vs. 14.5% women, *p* = 0.030). In the non-fasters group, regarding consumption of NOVA group 1 “unprocessed or minimally processed foods”, significantly more women were consuming legumes from three to six times per week (19.1% vs. 12.2% men, *p* = 0.038), fresh vegetables were consumed in quantities of at least one portion per day (52.4% vs. 43.2% men, *p* = 0.016), and traditional herbal tea was consumed at least once per day and more (12.7% vs. 4.1% men, *p* = 0.008). From the NOVA group 2 “processed culinary ingredients”, significantly more women consumed honey at least once per day and more (25.4% vs. 10.8% men, *p* = 0.003), whereas from the NOVA group 3 “processed foods”, significantly more women consumed tahini at least once per day and more (8.7% vs. 0% men, *p* = 0.008). Lastly, in terms of NOVA group 4 “UPFs” intake, significantly more women consumed marmalade at least once per day and more (4.8% vs. 0% men, *p* = 0.013) and traditional spoon desserts once per day (4.8% vs. 0% men, *p* = 0.008).

Fasters with MetS consumed biscuits and cakes with chocolate at least once per day more frequently (2.4% vs. 0% without MetS, *p* = 0.022) as well as cheese pastries three to six times per week more frequently (4.8% vs. 0% without MetS, *p* = 0.024). Among non-fasters, those with MetS consumed Cypriot pita bread one to two times per week significantly more often (19% vs. 6.5% without MetS, *p* = 0.023) and Coco Pops cereals three to six times per week (7.1% vs. 0% without MetS, *p* = 0.034).

With regard to socioeconomic status, fasters with low education consumed white cornflakes three to six times per week significantly more often (3.2% vs. 1.2% with higher education level, *p* < 0.001), consumed pizza one to three times per month significantly more often (4.8% vs. 3% with higher education level, *p* = 0.009), consumed full-fat fresh milk one to two times per week significantly more often (9.7% vs. 6.5% with higher education level, *p* = 0.016), consumed mustard one to two times per week significantly more often (9.7% vs. 0% with higher education level, *p* = 0.030), consumed round pita bread one to two times per week significantly more often (12.9% vs. 3.2% with higher education level, *p* = 0.040), consumed zero and/or light cola drinks three to six times per week significantly more often(3.2% vs. 1.7% with higher education level, *p* = 0.043), and consumed chocolate powder or beverage three to six times per week significantly more often (6.5% vs. 3.2% with higher education level, *p* = 0.047). Furthermore, fasters who were not married consumed pita bread once per day significantly more often (24.2% vs. 17.3% married, *p* = 0.001), consumed light fizzy drinks other than cola three to six times per week significantly more often (12.7% vs. 4.5% married, *p* = 0.002), consumed mustard three to six times per week significantly more often (28.5% vs. 13.7% married, *p* = 0.015), consumed round pita bread once per day significantly more often (22.7% vs. 6.5% married, (*p* = 0.019), consumed cornflakes with chocolate three to six times per week significantly more often (38.7% vs. 18.4% married, *p* = 0.026), and consumed ouzo, raki, and tsipouro one to two times per week significantly more often (3% vs. 0% married, *p* = 0.045). What is more, fasters who smoked consumed pizza three to six times per week significantly more often (8.5% vs. 2.7% non-smokers, *p* = 0.004), halvah with tahini once per day significantly more often (7.3% vs. 3.5% non-smokers, *p* = 0.046), and consumed ouzo, raki, and tsipouro three to six times per week significantly more often (12.5% vs. 3.7% non-smokers, *p* < 0.001).

Among non-fasters, no significant differences were observed in relation to educational status. Unmarried non-fasters consumed mustard three to six times per week significantly more often (12.3% vs. 7.4% married, *p* = 0.001), consumed desserts made with cholate once per day significantly more often (7.5% vs. 2.1% married, *p* = 0.016), and consumed light fizzy drinks other than cola one to two times per week significantly more often (2.5% v. 0% married, *p* = 0.022), while those who smoked consumed cheese pastries once per day significantly more often (2.5% vs. 0% non-smokers, *p* = 0.009), consumed traditional dips one to two times per week significantly more often (3.4% vs. 0% non-smokers, *p* = 0.017), and consumed yogurt with full fat three to six times per week significantly more often (2.7% vs. 0% non-smokers, *p* = 0.028).

A logistic regression model that included age, gender, education status, family status, smoking habits, body mass index (BMI) status, physical activity level, and energy intake was used to predict the probability of MetS presence. Fasters’ BMI status (*p* < 0.001) and physical activity levels (*p* = 0.006) were both significant individually and, more specifically, if fasters had higher BMI, they were ten times more likely to have MetS, and the higher the physical activity levels fasters had, the less likely MetS was to occur. In non-fasters, similarly, BMI status (*p* < 0.001) and physical activity levels (*p* = 0.017) were both significant individually, and, more specifically, if non-fasters had higher BMI, they were approximately five times more likely to have MetS; the more active non-fasters were, the less likely they were to have MetS. When analysis was performed on all participants, BMI status (*p* < 0.001) and physical activity levels (*p* < 0.001) were both significant individually, and, more specifically, if people had higher BMI, they were approximately six times more likely to have MetS; the more active they were, the less likely they were to have MetS. The results are presented in Table 3 for fasters, Table 4 for non-fasters, and Table 5 for all participants. In addition, another model that included all UPF items was used to investigate the same question in both and individual sample groups. No significant relations were found between MetS presence and UPF consumption (results in Supplemental Material Table S6).

## 4. Discussion

In the present study, the consumption of UPFs was compared between fasters and non-fasters during a non-fasting period. In terms of unprocessed or minimally processed foods, fasters consumed significantly less meat products (i.e., chicken, turkey, and beef) and more seafood, fish, soy products, and fresh fruits compared with non-fasters. This is in accordance with another study of 99 US COC faster adults who consumed significantly less red meat, poultry, processed meat, dairy products, and eggs during a fasting period [33]. Furthermore, a significantly higher intake of seafood, fish, and snails was reported among 10 Greek COC fasters when analysis was carried out in both fasting and non-fasting weeks [34].

To the best of our knowledge, this is the first study that focuses on the UPF consumption in a COC fasting population. In the present study, some socioeconomic factors were related to UPF intake among COC fasters. In more detail, among fasters, low education was significantly and positively associated with increased consumption of UPFs such as white cornflakes, pizza, full-fat milk, mustard, pita bread, light and/or zero cola drinks, and chocolate beverages. Furthermore, smoking significantly correlated with UPF intake, including pizza, halvah, ouzo, raki, and tsipouro. Similarly, unmarried fasters consumed pita breads, light fizzy drinks, mustard, pita bread, chocolate cornflakes, ouzo, raki, and tsipouro significantly more frequently. In accordance with our study, among a large population of 77,437 participants in the Adventist Health Study-2 (AHS-2), those with a higher intake of UPFs were of low education level, not married, smokers, had higher body mass index, and exercised less frequently compared with those with low consumption [35]. Similarly, in the French NutriNet-Santé cohort study that included 500 vegetarians, 254 vegans, 646 pesco-vegetarians, and 19,812 meat eaters, vegans consumed a higher proportion of UPFs (+6.41%) than meat eaters [36]. In the same study, the most frequently consumed products by vegans and vegetarians were plant-based meat and dairy substitutes such as plant-based drinks and soy protein foods [36]. Another analysis performed on 74,470 participants of the NutriNet-Santé cohort study showed that UPF intake was positively associated with lower educational level, smoking, and high BMI [37].

With regard to MetS, the present study found that both in fasters and non-fasters, those who had MetS consumed certain UPFs (i.e., chocolate biscuits, cakes, and cheese pastries in fasters; Cypriot bread and Coco Pops breakfast cereals in non-fasters) more often. Among 811 Canadian adults, a higher UPF intake was significantly related to a greater prevalence of MetS (adjusted odds ratio 1.90, 95% C.I. 1.14–3.17; *p* = 0.04) [38]. Furthermore, among 8065 participants of the Brazilian Longitudinal Study of Adult Health (ELSA-Brazil), every 150 gr daily increase in UPF consumption resulted in a 7% increased risk of MetS incidence [relative risk (RR) 1.07, 95% C.I. 1.05–1.08] [15]. In the same study, participants in the fourth quartile of UPF intake had a 33% increased risk of incident MetS compared with the first quartile (RR 1.33; 95% CI 1.20–1.47) [15]. In a previous analysis of the US National Health and Nutrition Examination Survey (NHANES 2009–2014) with 6385 participants, a higher UPF consumption (by 10%) was associated with an increased (by 4%) MetS prevalence [39]. Moreover, the participants with the highest UPF intake (i.e., >71%) correlated with a 28% greater prevalence of MetS compared with those with the lowest UPF consumption [39]. Similarly, among 789 adults from the general population in Tel Aviv, increased UPF intake was related to a 88% higher risk for MetS incidence (OR = 1.88, 95% C.I. 1.31–2.71; *p* = 0.001) as well as its individual components, i.e., hypertension (OR 1.53, 95% C.I. 1.07–2.19; *p* = 0.026), hypertriglyceridemia (OR 1.51, 95% C.I. 1.08–2.11; *p* = 0.017), and low HDL cholesterol (OR = 1.55, 95% C.I. 1.05–2.29; *p* = 0.028) [40]. A cross-sectional study with 302 Lebanese adults showed that the highest consumption of the “minimally processed/processed” dietary pattern was associated with significantly lower odds of MetS prevalence (OR 0.18, 95% CI 0.04–0.77), low HDL cholesterol (OR 0.17, 95% C.I. 0.05–0.60), and hyperglycemia (OR 0.25, 95% C.I. 0.07–0.98) [41]. In contrast, in a prospective study with 896 Brazilian adults from the 1978/79 Ribeirão Preto cohort, UPF intake had no association with MetS incidence; in women only, higher UPF consumption was related to a greater risk of abdominal obesity and low HDL cholesterol [42].

As COC religious fasting, among other fasting dietary patterns, is regarded a sustainable diet [43], future research with interventions that focus on sustainable healthy diets should also include this traditional aspect of the Mediterranean diet [44,45]. To the best of our knowledge, this is the first study to consider the consumption of UPFs among COC fasters and non-fasters as well as its association with MetS components and prevalence. Furthermore, the present data were gathered during a non-fasting period, thus representing the dietary habits of daily life. However, there are certain limitations similar to other epidemiological studies, like the collection of the self-reporting FFQ that could underestimate and/or overestimate someone’s dietary intake, and participants were volunteers; thus, a selection bias could exist.

## 5. Conclusions

Adopting a healthier diet, i.e., low in UPF intake and rich in unprocessed food consumption, throughout the year, and not only during fasting periods, may prevent MetS incidence among COC fasters. Certain socioeconomic factors, such as smoking, education, and marriage, may affect UPF consumption. Further studies assessing UPF intake in a larger sample size of adults who follow the COC fasting regime and in different countries are needed to elucidate the dietary habits and their potential health effects for this unique population during non-fasting periods.

## Figures and Tables

**Table 1:** Demographic and lifestyle habits of the participants.

Variable	Fasters (*n* = 200)	Non-Fasters (*n* = 200)	*p*-Value
	N (%)	N (%)	
**Sex**			0.60
Male	69 (34.5)	74 (37.0)	
Female	131 (65.5)	126 (63.0)	
**Education level**			0.17
Primary education	9 (4.5)	17 (8.5)	
Secondary education	51 (25.5)	54 (27.0)	
Tertiary education	113 (56.5)	104 (52.0)	
Master’s/doctoral	27 (13.5)	25 (12.5)	
**Marital status**			0.67
Single	88 (44.0)	94 (47.0)	
Married/living together	108 (54.0)	96 (48.0)	
Divorced	4 (2.0)	5 (2.5)	
Widowed	-	5 (2.5)	
**Smoking status**			0.000
Yes	11 (5.5)	58 (71.0)	
No—never	183 (91.5)	142 (29.0)	
No—quit smoking	6 (3.0)	-	
**Body Mass Index status**			0.15
Underweight (<18.5 kg/m^2^)	-	2 (1.0)	
Normal weight (18.5–24.9 kg/m^2^)	79 (39.5)	91 (45.5)	
Overweight (25–29.9 kg/m^2^)	76 (38.0)	68 (34.0)	
Obesity (≥30 kg/m^2^)	45 (22.5)	39 (19.5)	
**Physical activity level/status**			0.06
Extremely low (never/rarely)	12 (6.0)	27 (13.5)	
Low (<2 times per week)	57 (28.5)	53 (26.5)	
Moderate (2–3 timbmies per week)	98 (49.0)	94 (47.0)	
High (3–5 times per week)	30 (15.0)	22 (11.0)	
Extremely high (everyday)	3 (1.5)	4 (2.0)	
**Free-time workout**			0.26
Yes	74 (37.0)	85 (42.5)	
No	126 (63.0)	115 (57.5)	
	**Mean ± SD**	**Mean ± SD**	***p*-value**
**Age (years)**	43.4 ± 16.7	41.9 ± 17.3	0.40
**Frequency of free-time workouts (times/week)**	3.4 ± 2.0	4.1 ± 1.9	0.021
**Duration of free-time workouts (hours/activity)**	1.2 ± 0.7	1.2 ± 0.7	0.81
**Total duration of free-time workouts (hours/week)**	1.6 ± 3.4	2.1 ± 3.5	0.18
**Sleeping (hours/night)**	6.8 ± 1.2	6.9 ± 1.2	0.23
**Sleeping (hours/day)**	0.8 ± 0.9	1.2 ± 1.4	0.008
**Watching TV (hours/day)**	1.1 ± 1.3	2.2 ± 1.7	0.000
**Screen time (PC/mobile) (hours/day)**	2.3 ± 2.9	2.9 ± 2.9	0.043
**Reading (hours/day)**	1.4 ± 1.2	1.3 ± 1.4	0.16

**Table 2:** Classification of food items regarding the extent of processing (NOVA classification).

NOVA Food Category	Food Items *
Group 1: Unprocessed or minimally processed foods	Beef, chicken, Greek coffee, Nescafe, filtered coffee, espresso, corn, egg, fish fresh, dried fruit, fresh fruit, fresh fruit juice, lamb and goat, legumes, fresh non-fat milk, fresh low-fat (1.5%) milk, milk goat, milk products from soya, nut barley, nuts, white pasta, whole grain pasta, pork, boiled potato, roasted potato, rabbit, white rice, whole grain rice, seafood, shellfish, snail, soya, black tea, green tea, herbal traditional tea, traxana and couscous, turkey, low-fat cow yogurt (2%), non-fat cow yogurt, goat yogurt, boiled vegetable, fresh vegetable, water.
Group 2: Processed culinary ingredients	Butter, honey, oilseed, olive oil, sugar.
Group 3: Processed foods	Bagel and koulouri, beer, white bread, whole grain bread, breadsticks, breadsticks with seeds, 12%-fat cheese, mizithra, anthotiro, low-fat cheese, cottage, low-fat cheese like Milner, Fina, cheese products from soya, whole fat cheese, feta, kasseri, kefalotiri, graviera, parmezana, canned fish, canned fruit, canned fruit juice, olive, pastitsio/mousaka, homemade pies, fried potato, traditional rusk, wheat rusk, whole grain rusk, stuffed peppers, grape leaves, cabbage, spinach with rice, cabbage with rice and meat, tahini, pickled vegetable, cooked vegetables (ladera), wine.
Group 4: Ultra-processed foods	Stuffed bagel koulouri, bars, Becel, plain biscuits/cake, biscuits/cake with chocolate, cacao powder, potato chips, chocolate powder or beverage, Coco Pops, white cornflakes, whole grain cornflakes, cornflakes with chocolate, homemade dessert, spoon dessert (koutaliou), dessert with chocolate, donut and loukoumas, energy drinks, fizzy drink, cola light zero, fizzy cola drink with sugar, other fizzy drink light, other fizzy drink with sugar, halvah semolina, halvah tahini, ice cream, isotonic drinks, ketchup, lemonade/orangeade with sugar, margarine, marmalade, mayonnaise, light mayonnaise, non-fat milk chocolate beverage, whole milk chocolate beverage, condensed full-fat milk, full-fat fresh milk (3.5%), muesli oats, mustard, nougat, nougat with nuts, ouzo, raki and tsipouro, peanut butter, pita bread, Cypriot pita bread, round pita bread, pizza, full-fat milk or rice pudding, salad dip (traditional), tirokauteri, melitzanosalata, pork sausage, turkey sausage, spirit beverages, tarama, full-fat cow yogurt (10%), dessert yogurt.

* Food items are presented in alphabetical order.

**Table 3:** Logistic regression analysis results for fasters.

Variables in the Equation									
		B	S.E.	Wald	df	Sig.	Exp(B)	95% C.I. for Exp(B)	
								Lower	Upper
Step 1 ^a^	Gender	0.045	0.594	0.006	1	0.940	1.046	0.326	3.350
	Age	−0.001	0.022	0.001	1	0.974	0.999	0.958	1.043
	Education_status	−0.695	0.371	3.517	1	0.061	0.499	0.241	1.032
	Family_status	−0.678	0.634	1.143	1	0.285	0.508	0.147	1.760
	Smoking_status	−0.523	0.582	0.810	1	0.368	0.592	0.189	1.853
	BMI_status	2.362	0.484	23.803	1	0.000	10.612	4.109	27.409
	PA_status	−1.039	0.375	7.654	1	0.006	0.354	0.170	0.739
	Total_energy_kcal	−0.001	0.001	0.957	1	0.328	0.999	0.998	1.001
	Constant	−2.701	3.095	0.761	1	0.383	0.067		

^a^ Variable(s) entered in step 1: Gender, Age, Education_status, Family_status, Smoking_status, BMI_status, PA_status, Total_energy_kcal.

**Table 4:** Logistic regression analysis results for non-fasters.

Variables in the Equation									
		B	S.E.	Wald	df	Sig.	Exp(B)	95% C.I. for Exp(B)	
								Lower	Upper
Step 1 ^a^	Gender	−0.760	0.469	2.629	1	0.105	0.468	0.187	1.172
	Age	0.033	0.022	2.251	1	0.133	1.033	0.990	1.078
	Education_status	0.104	0.286	0.132	1	0.717	1.109	0.634	1.942
	Family_status	−0.185	0.566	0.106	1	0.744	0.831	0.274	2.521
	Smoking_status	−0.057	0.461	0.015	1	0.901	0.944	0.383	2.331
	BMI_status	1.563	0.331	22.325	1	0.000	4.772	2.496	9.126
	PA_status	−0.621	0.259	5.723	1	0.017	0.538	0.323	0.894
	Total_energy_kcal	0.000	0.000	0.005	1	0.944	1.000	0.999	1.001
	Constant	−4.823	1.993	5.855	1	0.016	0.008		

^a^ Variable(s) entered in step 1: Gender, Age, Education_status, Family_status, Smoking_status, BMI_status, PA_status, Total_energy_kcal.

**Table 5:** Logistic regression analysis results for all participants.

Variables in the Equation									
		B	S.E.	Wald	df	Sig.	Exp(B)	95% C.I. for Exp(B)	
								Lower	Upper
Step 1 ^a^	Gender	−0.409	0.345	1.405	1	0.236	0.664	0.338	1.307
	Age	0.018	0.014	1.665	1	0.197	1.019	0.991	1.047
	Education_status	−0.258	0.210	1.506	1	0.220	0.773	0.512	1.167
	Family_status	−0.300	0.392	0.587	1	0.444	0.741	0.344	1.596
	Smoking_status	−0.096	0.346	0.078	1	0.780	0.908	0.461	1.788
	BMI_status	1.740	0.255	46.400	1	0.000	5.699	3.454	9.403
	PA_status	−0.757	0.202	13.982	1	0.000	0.469	0.316	0.698
	Total_energy_kcal	0.000	0.000	0.010	1	0.922	1.000	0.999	1.001
	Constant	−4.080	1.603	6.480	1	0.011	0.017		

^a^ Variable(s) entered in step 1: Gender, Age, Education_status, Family_status, Smoking_status, BMI_status, PA_status, Total_energy_kcal.

## Data Availability

The data presented in this study are available on request from the corresponding author.

## Supplementary Materials

The following supporting information can be downloaded at: https://www.mdpi.com/article/10.3390/nu15234907/s1, Table S1: Total food items considered in the present cross-sectional study according to the extent of processing (NOVA classification) in alphabetical order; Table S2: Intake of unprocessed or minimally processed foods based on food frequency questionnaire in fasters and non-fasters; Table S3: Intake of processed culinary ingredients based on food frequency questionnaire in fasters and non-fasters; Table S4: Intake of processed foods based on food frequency questionnaire in fasters and non-fasters; Table S5: Intake of ultra-processed foods based on food frequency questionnaire in fasters and non-fasters; Table S6: Logistic regression analysis for UPF food items.
