# Supplementary material for: Consumption of Ultra-Processed Food and Drink Products in a Greek Christian Orthodox Church Fasting Population
Source: Nutrients. 2023 Nov 24;15(23):4907. doi: 10.3390/nu15234907 (PMC10708049; doi:10.3390/nu15234907)
Supplement: Supplementary file 1 [file nutrients-15-04907-s001.zip › nutrients-2648915-supplementary.pdf]

Supplementary Material

Table S1. Total food items considered in the present cross-sectional study according to the extent of processing (NOVA classification) in alphabetical order

NOVA classification	Food groups	Food categories
1	Beef	Meat and meat products
1	Chicken	Meat and meat products
1	Coffee Greek, nescafe, filtered, espresso	Non-alcoholic beverages
1	Corn	Cereals
1	Egg	Eggs and egg products
1	Fish fresh	Fish and fish products
1	Fruit dried	Fruits and fruit products
1	Fruit fresh	Fruits and fruit products
1	Fruit juice fresh	Fruits and fruit products
1	Lamb and goat	Meat and meat products
1	Legumes	Legumes and legumes products
1	Milk fresh nonfat	Milk and dairy products
1	Milk fresh low fat 1.5%	Milk and dairy products
1	Milk goat	Milk and dairy products
1	Milk products from soya	Legumes and legume products
1	Nut barley	Cereals
1	Nuts	Nuts
1	Pasta white	Cereals
1	Pasta whole grain	Cereals
1	Pork	Meat and meat products
1	Potato boiled	Tubers and tuber products
1	Potato roasted	Tubers and tuber products
1	Rabbit	Meat and meat products
1	Rice white	Cereals
1	Rice whole grain	Cereals
1	Seafood	Seafood
1	Shellfish	Seafood
1	Snail	Seafood
1	Soya	Legumes and legume products
1	Tea black	Non-alcoholic beverages
1	Tea green	Non-alcoholic beverages
1	Tea herbal traditional	Non-alcoholic beverages
1	Traxanas, couscous	Cereals
1	Turkey	Meat and meat products

1	Yogurt cow low fat 2%	Milk and dairy products
1	Yogurt cow nonfat	Milk and dairy products
1	Yogurt goat	Milk and dairy products
1	Vegetable boiled	Vegetables
1	Vegetable fresh	Vegetables
1	Water	Non-alcoholic beverages
2	Butter	Oils and fats
2	Honey	Sugars and sugary products
2	Oilseed	Oils and fats
2	Olive oil	Oils and fats
2	Sugar	Sugars and sugary products
3	Bagel, koulouri	Cereals
3	Beer	Alcoholic beverages
3	Bread white	Cereals
3	Bread wholegrain	Cereals
3	Breadsticks	Cereals
3	Breadsticks with seeds	Cereals
3	Cheese 12% fat, mizithra, anthotiro	Milk and dairy products
3	Cheese low fat, cottage	Milk and dairy products
3	Cheese low fat, ex. milner, fina	Milk and dairy products
3	Cheese products from soya	Milk and dairy products
3	Cheese whole fat, feta, kasseri, kefalotiri	Milk and dairy products
3	Cheese whole fat, graviera, parmezana	Milk and dairy products
3	Fish canned	Fish and fish products
3	Fruit canned	Fruits and fruit products
3	Fruit juice canned	Fruits and fruit products
3	Olive	Oils and fats
3	Pastitsio and moussaka	Homemade cooked food
3	Pies homemade	Homemade cooked food
3	Potato fried	Tubers and tuber products
3	Rusk traditional	Cereals
3	Rusk wheat	Cereals
3	Rusk whole grain	Cereals
3	Stuffed peppers grape leaves cabbage spinach with rice	Homemade cooked food
3	Stuffed peppers grape leaves cabbage with rice and meat	Homemade cooked food
3	Tahini	Nuts
3	Vegetable pickled	Vegetables
3	Vegetables cooked, including ladera	Homemade cooked food
3	Wine	Alcoholic beverages
4	Bagel koulouri stuffed	Cereals

4	Bars	Sugars and sugary products
4	Becel margarine	Oils and fats
4	Biscuits cake plain	Sugars and sugary products
4	Biscuits cake with chocolate	Sugars and sugary products
4	Cacao powder	Non-alcoholic beverages
4	Chips potato	Salty snacks
4	Chocolate powder or beverage	Non-alcoholic beverages
4	Coco pops	Cereals
4	Corn flakes white	Cereals
4	Corn flakes whole grain	Cereals
4	Corn flakes with chocolate	Cereals
4	Dessert homemade, dessert spoon	Sugars and sugary products
4	Dessert with chocolate	Sugars and sugary products
4	Donut and loukoumas	Sugars and sugary products
4	Energy drinks	Non-alcoholic beverages
4	Fizzy drink cola light zero	Non-alcoholic beverages
4	Fizzy drink cola with sugar	Non-alcoholic beverages
4	Fizzy drink light other	Non-alcoholic beverages
4	Fizzy drink with sugar other	Non-alcoholic beverages
4	Halvah semolina	Sugars and sugary products
4	Halvah tahini	Sugars and sugary products
4	Ice cream	Sugars and sugary products
4	Isotonic drinks	Non-alcoholic beverages
4	Ketchup	Sauces and dressings
4	Lemonade orangeade with sugar	Non-alcoholic beverages
4	Margarine	Oils and fats
4	Marmalade	Sugars and sugary products
4	Mayonnaise	Sauces and dressings
4	Mayonnaise light	Sauces and dressings
4	Milk chocolate beverage nonfat	Milk and dairy products
4	Milk chocolate beverage whole milk	Milk and dairy products
4	Milk condensed full fat	Milk and dairy products
4	Milk fresh full fat 3.5%	Milk and dairy products
4	Muesli oats	Cereals
4	Mustard	Sauces and dressings
4	Nougat	Sugars and sugary products
4	Nougat with nuts	Sugars and sugary products
4	Ouzo, raki, tsipouro	Alcoholic beverages
4	Peanut butter	Nuts
4	Pita bread	Cereals
4	Pita bread Cypriot	Cereals

4	Pita bread round	Cereals
4	Pizza	Pre-cooked dishes
4	Pudding milk or rice full fat	Cereals
4	Salad dip (traditional), including tirokauteri, melitzanosalata	Sauces and dressings
4	Sausage pork	Meat and meat products
4	Sausage turkey	Meat and meat products
4	Spirit beverages	Alcoholic beverages
4	Tarama	Sauces and dressings
4	Yogurt cow full fat 10%	Milk and dairy products
4	Yogurt dessert	Milk and dairy products

Table S2. Intake of unprocessed or minimally processed foods based on food frequency questionnaire in fasters and non-fasters.

Variables	Fasters (n=174)	Non-fasters (n=176)	p-value
	n (%)	n (%)	
Pork			0.75
Never/rarely	42 (21.0)	26 (13.0)	
1-3 times/month	61 (30.5)	89 (44.5)	
1-2 times/week	65 (32.5)	56 (28.0)	
3-6 times/week	6 (3.0)	5 (2.5)	
1 time/day	0	0	
>2 times/day	0	0	
Beef			0.007
Never/rarely	33 (16.5)	12 (6.0)	
1-3 times/month	86 (43.0)	97 (48.5)	
1-2 times/week	52 (26.0)	59 (29.5)	
3-6 times/week	2 (1.0)	8 (4.0)	
1 time/day	1 (0.5)	0	
>2 times/day	0	0	
Goat, lamb			0.30
Never/rarely	164 (82.0)	161 (80.5)	
1-3 times/month	1 (0.5)	1 (0.5)	
1-2 times/week	9 (4.5)	14 (7.0)	
3-6 times/week	0	0	
1 time/day	0	0	
>2 times/day	0	0	
Chicken			0.001
Never/rarely	30 (15.0)	10 (5.0)	
1-3 times/month	108 (54.0)	113 (56.5)	
1-2 times/week	29 (14.5)	37 (18.5)	
3-6 times/week	7 (3.5)	15 (7.5)	
1 time/day	0	1 (0.5)	

>2 times/day	0	0	
Rabbit			0.38
Never/rarely	171 (85.5)	170 (85.0)	
1-3 times/month	0	1 (0.5)	
1-2 times/week	3 (1.5)	5 (2.5)	
3-6 times/week	0	0	
1 time/day	0	0	
>2 times/day	0	0	
Turkey			0.003
Never/rarely	171 (85.5)	159 (79.5)	
1-3 times/month	1 (0.5)	7 (3.5)	
1-2 times/week	2 (1.0)	8 (4.0)	
3-6 times/week	0	1 (0.5)	
1 time/day	0	1 (0.5)	
>2 times/day	0	0	
Fish fresh			0.18
Never/rarely	11 (5.5)	23 (11.5)	
1-3 times/month	113 (56.5)	102 (51.0)	
1-2 times/week	46 (23.0)	51 (25.5)	
3-6 times/week	4 (2.0)	0	
1 time/day	0	0	
>2 times/day	0	0	
Seafood			0.008
Never/rarely	1 (0.5)	50 (25.0)	
1-3 times/month	59 (29.5)	87 (43.5)	
1-2 times/week	109 (37.5)	63 (31.5)	
3-6 times/week	5 (32.5)	0	
1 time/day	0	0	
>2 times/day	0	0	
Shellfish			0.075
Never/rarely	133 (66.5)	172 (86.0)	
1-3 times/month	5 (2.5)	3 (1.5)	
1-2 times/week	35 (17.5)	25 (12.5)	
3-6 times/week	1 (0.5)	0	
1 time/day	0	0	
>2 times/day	0	0	
Snails			0.026
Never/rarely	165 (82.5)	198 (99.0)	
1-3 times/month	0	2 (1.0)	
1-2 times/week	7 (3.5)	0	
3-6 times/week	2 (1.0)	0	
1 time/day	0	0	
>2 times/day	0	0	
Egg			0.51
Never/rarely	15 (7.5)	20 (10.0)	
1-3 times/month	82 (41.0)	74 (37.0)	

1-2 times/week	30 (15.0)	46 (23.0)	
3-6 times/week	38 (19.0)	24 (12.0)	
1 time/day	5 (2.5)	12 (6.0)	
>2 times/day	4 (2.0)	0	
Legumes			0.67
Never/rarely	5 (2.5)	6 (3.0)	
1-3 times/month	142 (71.0)	137 (68.5)	
1-2 times/week	14 (7.0)	29 (14.5)	
3-6 times/week	13 (6.5)	4 (2.0)	
1 time/day	0	0	
>2 times/day	0	0	
Soya			0.001
Never/rarely	146 (73.0)	163 (81.5)	
1-3 times/month	8 (4.0)	10 (5.0)	
1-2 times/week	19 (9.5)	3 (1.5)	
3-6 times/week	1 (0.5)	0	
1 time/day	0	0	
>2 times/day	0	0	
Milk goat			0.39
Never/rarely	165 (82.5)	172 (86.0)	
1-3 times/month	2 (1.0)	0	
1-2 times/week	2 (1.0)	0	
3-6 times/week	3 (1.5)	2 (1.0)	
1 time/day	2 (1.0)	2 (1.0)	
>2 times/day	0	0	
Milk fresh nonfat			0.75
Never/rarely	168 (84.0)	169 (84.5)	
1-3 times/month	1, 0.5)	1 (0.5)	
1-2 times/week	3 (1.5)	2 (1.0)	
3-6 times/week	1 (0.5)	4 (2.0)	
1 time/day	1 (0.5)	0	
>2 times/day	0	0	
Milk fresh low fat, up to 1.5%			0.081
Never/rarely	116 (58.0)	108 (54.0)	
1-3 times/month	15 (7.5)	11 (5.5)	
1-2 times/week	9 (4.5)	9 (4.5)	
3-6 times/week	22 (11.0)	26 (13)	
1 time/day	12 (6.0)	20 (10.0)	
>2 times/day	0	2 (1.0)	
Milk products from soya			0.87
Never/rarely	170 (85.0)	172 (86.0)	
1-3 times/month	0	0	
1-2 times/week	3 (1.5)	3 (1.5)	
3-6 times/week	1 (0.5)	0	
1 time/day	0	0	
>2 times/day	0	1 (0.5)	

Yogurt cow nonfat			0.26
Never/rarely	164 (82.0)	172 (86.0)	
1-3 times/month	4 (2.0)	1 (0.5)	
1-2 times/week	5 (2.5)	1 (0.5)	
3-6 times/week	1 (0.5)	2 (1.0)	
1 time/day	0	0	
>2 times/day	0	0	
Yogurt cow low fat, up to 2%			0.50
Never/rarely	90 (45.0)	94 (47.0)	
1-3 times/month	23 (11.5)	31 (15.5)	
1-2 times/week	30 (15.0)	22 (11.0)	
3-6 times/week	23 (11.5)	20 (10.0)	
1 time/day	7 (3.5)	9 (4.5)	
>2 times/day	1 (0.5)	0	
Yogurt goat			0.33
Never/rarely	122 (61.0)	135 (67.5)	
1-3 times/month	28 (14.0)	17 (8.5)	
1-2 times/week	10 (5.0)	15 (7.5)	
3-6 times/week	12 (6.0)	6 (3.0)	
1 time/day	1 (0.5)	3 (1.5)	
>2 times/day	1 (0.5)	0	
Nut barley			0.96
Never/rarely	138 (69.0)	143 (71.5)	
1-3 times/month	15 (7.5)	11 (5.5)	
1-2 times/week	14 (7.0)	14 (7.0)	
3-6 times/week	5 (2.5)	5 (2.5)	
1 time/day	2 (1.0)	3 (1.5)	
>2 times/day	0	0	
Pasta white			0.40
Never/rarely	15 (7.5)	20 (10.0)	
1-3 times/month	131 (65.5)	115 (57.5)	
1-2 times/week	18 (9.0)	28 (14.0)	
3-6 times/week	10 (5.0)	13 (6.5)	
1 time/day	0	0	
>2 times/day	0	0	
Pasta whole grain			0.16
Never/rarely	150 (75.0)	145 (72.5)	
1-3 times/month	14 (7.0)	14 (7.0)	
1-2 times/week	10 (5.0)	15 (7.5)	
3-6 times/week	0	2 (1.0)	
1 time/day	0	0	
>2 times/day	0	0	
Potato boiled			0.65
Never/rarely	60 (30.0)	55 (27.5)	
1-3 times/month	41 (20.5)	47 (23.5)	
1-2 times/week	68 (34.0)	67 (33.5)	

3-6 times/week	5 (2.5)	7 (3.5)	
1 time/day	0	0	
>2 times/day	0	0	
Potato roasted			0.41
Never/rarely	19 (9.5)	17 (8.5)	
1-3 times/month	96 (48.0)	90 (45.0)	
1-2 times/week	50 (25.0)	59 (29.5)	
3-6 times/week	8 (4.0)	10 (5.0)	
1 time/day	1 (0.5)	0	
>2 times/day	0	0	
Rice white			0.91
Never/rarely	16 (8.0)	26 (13.0)	
1-3 times/month	104 (52.0)	83 (41.5)	
1-2 times/week	46 (23.0)	59 (29.5)	
3-6 times/week	7 (3.5)	8 (4.0)	
1 time/day	1 (0.5)	0	
>2 times/day	0	0	0.89
Rice wholegrain			
Never/rarely	146 (73.0)	151 (75.5)	
1-3 times/month	15 (7.5)	10 (5.0)	
1-2 times/week	13 (6.5)	15 (7.5)	
3-6 times/week	0	0	
1 time/day	0	0	
>2 times/day	0	0	
Traxana and couscous			0.28
Never/rarely	100 (50.0)	112 (56.0)	
1-3 times/month	23 (11.5)	21 (10.5)	
1-2 times/week	49 (24.5)	40 (20.0)	
3-6 times/week	1 (0.5)	2 (1.0)	
1 time/day	1 (0.5)	1 (0.5)	
>2 times/day	0	0	
Corn			0.22
Never/rarely	126 (63.0)	135 (67.5)	
1-3 times/month	7 (3.5)	11 (5.5)	
1-2 times/week	39 (19.5)	28 (14.0)	
3-6 times/week	2 (1.0)	2 (1.0)	
1 time/day	0	0	
>2 times/day	0	0	
Nuts			0.87
Never/rarely	44 (22.0)	43 (21.5)	
1-3 times/month	45 (22.5)	46 (23.0)	
1-2 times/week	34 (17.0)	34 (17.0)	
3-6 times/week	18 (9.0)	26 (13.0)	
1 time/day	20 (10.0)	16 (8.0)	
>2 times/day	13 (6.5)	11 (5.5)	
Vegetables fresh			0.43

Never/rarely	5 (2.5)	23 (11.5)	
1-3 times/month	18 (9.0)	13 (6.5)	
1-2 times/week	5 (2.5)	42 (21.0)	
3-6 times/week	43 (21.5)	79 (39.5)	
1 time/day	75 (37.5)	19 (9.5)	
>2 times/day	28 (14.0)	0	
Vegetables fresh, boiled			0.60
Never/rarely	28 (14.0)	30 (15.0)	
1-3 times/month	40 (20.0)	46 (23.0)	
1-2 times/week	30 (15.0)	23 (11.5)	
3-6 times/week	38 (19.0)	41 (20.5)	
1 time/day	29 (14.5)	30 (15.0)	
>2 times/day	9 (4.5)	6 (3.0)	
Fruit fresh			0.022
Never/rarely	2 (1.0)	7 (3.5)	
1-3 times/month	21 (10.5)	27 (13.5)	
1-2 times/week	4 (2.0)	11 (5.5)	
3-6 times/week	27 (13.5)	27 (13.5)	
1 time/day	52 (26.0)	48 (24.0)	
>2 times/day	68 (34.0)	56 (28.0)	
Fruit juice fresh			0.46
Never/rarely	74 (37.0)	83 (41.5)	
1-3 times/month	36 (18.0)	31 (15.5)	
1-2 times/week	32 (16.0)	36 (18.0)	
3-6 times/week	15 (7.5)	11 (5.5)	
1 time/day	16 (8.0)	13 (6.5)	
>2 times/day	1 (0.5)	2 (1.0)	
Fruit dried			0.06
Never/rarely	105 (52.5)	121 (60.5)	
1-3 times/month	18 (9.0)	16 (8.0)	
1-2 times/week	28 (14.0)	25 (12.5)	
3-6 times/week	12 (6.0)	7 (3.5)	
1 time/day	6 (3.0)	5 (2.5)	
>2 times/day	5 (2.5)	2 (1.0)	
Coffee including Greek, filtered, nescafe and espresso			0.69
Never/rarely	7 (3.5)	2 (1.0)	
1-3 times/month	53 (26.5)	47 (23.5)	
1-2 times/week	14 (7.0)	11 (5.5)	
3-6 times/week	7 (3.5)	7 (3.5)	
1 time/day	62 (31.0)	73 (36.5)	
>2 times/day	31 (15.5)	36 (18.0)	
Tea black			0.63
Never/rarely	147 (73.5)	149 (74.5)	
1-3 times/month	7 (3.5)	7 (3.5)	
1-2 times/week	15 (7.5)	10 (5.0)	

3-6 times/week	5 (2.5)	7 (3.5)	
1 time/day	0	3 (1.5)	
>2 times/day	0	0	
Tea green			0.81
Never/rarely	109 (54.5)	115 (57.5)	
1-3 times/month	23 (11.5)	18 (9.0)	
1-2 times/week	21 (10.5)	18 (9.0)	
3-6 times/week	11 (5.5)	17 (8.5)	
1 time/day	6 (3.0)	7 (3.5)	
>2 times/day	4 (2.0)	1 (0.5)	
Tea herbal, traditional			0.83
Never/rarely	82 (41.0)	89 (44.5)	
1-3 times/month	29 (14.5)	24 (12.0)	
1-2 times/week	32 (16.0)	36 (18.0)	
3-6 times/week	17 (8.5)	8 (4.0)	
1 time/day	12 (6.0)	16 (8.0)	
>2 times/day	2 (1.0)	3 (1.5)	
Water			0.10
Never/rarely	0	0	
1-3 times/month	0	0	
1-2 times/week	0	0	
3-6 times/week	0	0	
1 time/day	6 (3.0)	13 (6.5)	
>2 times/day	168 (84.0)	163 (81.5)	

Table S3. Intake of processed culinary ingredients based on food frequency questionnaire in fasters and non-fasters.

Variables	Fasters (n=174)	Non-fasters (n=176)	p-value
	n (%)	n (%)	
Butter			0.68
Never/rarely	119 (59.5)	120 (60)	
1-3 times/month	20 (10.0)	22 (11.0)	
1-2 times/week	30 (15.0)	22 (11.0)	
3-6 times/week	2 (1.0)	6 (3.0)	
1 time/day	1 (0.5)	5 (2.5)	
>2 times/day	2 (1.0)	1 (0.5)	
Honey			0.56
Never/rarely	37 (18.5)	40 (20.0)	
1-3 times/month	43 (21.5)	39 (19.5)	
1-2 times/week	28 (14.0)	30 (15.0)	
3-6 times/week	33 (16.5)	27 (13.5)	
1 time/day	27 (13.5)	23 (11.5)	
>2 times/day	6 (3.0)	17 (8.5)	
Oilseed			0.54

Never/rarely	98 (49.0)	94 (47.0)	
1-3 times/month	26 (13.0)	32 (16.0)	
1-2 times/week	37 (18.5)	30 (15.0)	
3-6 times/week	7 (3.5)	14 (7.0)	
1 time/day	5 (2.5)	3 (1.5)	
>2 times/day	1 (0.5)	3 (1.5)	
Olive oil			0.11
Never/rarely	13 (6.5)	23 (11.5)	
1-3 times/month	7 (3.5)	4 (2.0)	
1-2 times/week	6 (3.0)	9 (4.5)	
3-6 times/week	7 (3.5)	8 (4.0)	
1 time/day	15 (7.5)	18 (9.0)	
>2 times/day	126 (63.0)	114 (57.0)	
Sugar			0.95
Never/rarely	44 (22.0)	49 (24.5)	
1-3 times/month	20 (10.0)	19 (9.5)	
1-2 times/week	11 (5.5)	10 (5.0)	
3-6 times/week	16 (8.0)	20 (10.0)	
1 time/day	39 (19.5)	20 (10.0)	
>2 times/day	44 (22.0)	58 (29.0)	

Table S4. Intake of processed foods based on food frequency questionnaire in fasters and non-fasters.

Variables	Fasters (n=174)	Non-fasters (n=176)	p-value
	n (%)	n (%)	
Fish canned and smoked			0.23
Never/rarely	136 (68.0)	143 (71.5)	
1-3 times/month	4 (2.0)	11 (5.5)	
1-2 times/week	34 (17.0)	21 (10.5)	
3-6 times/week	0	1 (0.5)	
1 time/day	0	0	
>2 times/day	0	0	
Cheese whole fat, including feta, kasseri, kefalotiri			0.71
Never/rarely	12 (6.0)	19 (9.5)	
1-3 times/month	32 (16.0)	29 (14.5)	
1-2 times/week	8 (4.0)	4 (2.0)	
3-6 times/week	72 (36.0)	61 (30.5)	
1 time/day	31 (15.5)	36 (18.0)	
>2 times/day	19 (9.5)	27 (13.5)	
Cheese whole fat, including graviera, parmesan			0.88
Never/rarely	77 (38.5)	80 (40.0)	
1-3 times/month	37 (18.5)	40 (20.0)	
1-2 times/week	42 (21.0)	40 (20.0)	

3-6 times/week	15 (7.5)	8 (4.0)	
1 time/day	2 (1.0)	6 (3.0)	
>2 times/day	1 (0.5)	2 (1.0)	
Cheese 12% fat, including mizithra, anthotiro			0.58
Never/rarely	131 (65.5)	135 (67.5)	
1-3 times/month	11 (5.5)	16 (8.0)	
1-2 times/week	20 (10.0)	14 (7.0)	
3-6 times/week	9 (4.5)	8 (4.0)	
1 time/day	3 (1.5)	2 (1.0)	
>2 times/day	0	1 (0.5)	
Cheese low fat, including milner, fina			0.21
Never/rarely	140 (70.0)	131 (65.5)	
1-3 times/month	9 (4.5)	16 (8.0)	
1-2 times/week	13 (6.5)	10 (5.0)	
3-6 times/week	8 (4.0)	10 (5.0)	
1 time/day	2 (1.0)	7 (3.5)	
>2 times/day	2 (1.0)	2 (1.0)	
Cheese low fat, only cottage			0.74
Never/rarely	159 (79.5)	159 (79.5)	
1-3 times/month	2 (1.0)	3 (1.5)	
1-2 times/week	6 (3.0)	11 (5.5)	
3-6 times/week	4 (2.0)	2 (1.0)	
1 time/day	2 (1.0)	1 (0.5)	
>2 times/day	1 (0.5)	0	
Cheese products from soya			0.23
Never/rarely	146 (73.0)	158 (79.0)	
1-3 times/month	9 (4.5)	6 (3.0)	
1-2 times/week	18 (9.0)	9 (4.5)	
3-6 times/week	1 (0.5)	2 (1.0)	
1 time/day	0	1 (0.5)	
>2 times/day	0	0	
Bread white			0.50
Never/rarely	37 (18.5)	42 (21.0)	
1-3 times/month	20 (10.0)	12 (6.0)	
1-2 times/week	15 (7.5)	13 (6.5)	
3-6 times/week	17 (8.5)	16 (8.0)	
1 time/day	33 (16.5)	27 (13.5)	
>2 times/day	52 (26.0)	66 (33.0)	
Bread whole grain			0.25
Never/rarely	58 (29.0)	76 (38.0)	
1-3 times/month	21 (10.5)	14 (7.0)	
1-2 times/week	12 (6.0)	11 (5.5)	
3-6 times/week	13 (6.5)	15 (7.5)	
1 time/day	30 (15.0)	17 (8.5)	

>2 times/day	40 (20.0)	43 (21.5)	
Breadsticks			0.73
Never/rarely	125 (62.5)	136 (68.0)	
1-3 times/month	18 (9.0)	8 (4.0)	
1-2 times/week	27 (13.5)	26 (13.0)	
3-6 times/week	1 (0.5)	3 (1.5)	
1 time/day	3 (1.5)	2 (1.0)	
>2 times/day	0	1 (0.5)	
Breadsticks with seeds			0.08
Never/rarely	122 (61.0)	140 (70.0)	
1-3 times/month	15 (7.5)	11 (5.5)	
1-2 times/week	31 (15.5)	21 (10.5)	
3-6 times/week	3 (1.5)	2 (1.0)	
1 time/day	3 (1.5)	0	
>2 times/day	0	2 (1.0)	
Bagel and koulouri			0.43
Never/rarely	65 (32.5)	68 (34.0)	
1-3 times/month	38 (19.0)	48 (24.0)	
1-2 times/week	43 (21.5)	32 (16.0)	
3-6 times/week	16 (8.0)	17 (8.5)	
1 time/day	9 (4.5)	11 (5.5)	
>2 times/day	3 (1.5)	0	
Rusk traditional			0.89
Never/rarely	133 (66.5)	132 (66)	
1-3 times/month	12 (6.0)	15 (7.5)	
1-2 times/week	24 (12.0)	24 (12)	
3-6 times/week	4 (2.0)	4 (2)	
1 time/day	1 (0.5)	1 (0.5)	
>2 times/day	0	0	
Rusk wheat			0.20
Never/rarely	128 (64.0)	141 (70.5)	
1-3 times/month	14 (7.0)	10 (.05)	
1-2 times/week	21 (10.5)	19 (9.5)	
3-6 times/week	5 (2.5)	1 (0.5)	
1 time/day	3 (1.5)	2 (1.0)	
>2 times/day	3 (1.5)	3 (1.5)	
Rusk whole grain			0.69
Never/rarely	135 (67.5)	137 (68.5)	
1-3 times/month	14 (7.0)	7 (3.5)	
1-2 times/week	19 (9.5)	25 (12.5)	
3-6 times/week	2 (1.0)	3 (1.5)	
1 time/day	3 (1.5)	3 (1.5)	
>2 times/day	1 (0.5)	1 (0.5)	
Pastitsio and moussaka			0.11
Never/rarely	78 (39.0)	64 (32.0)	
1-3 times/month	11 (5.5)	12 (6.0)	

1-2 times/week	85 (42.5)	100 (50.0)	
3-6 times/week	0	0	
1 time/day	0	0	
>2 times/day	0	0	
Pies homemade			0.31
Never/rarely	30 (15.0)	38 (19.0)	
1-3 times/month	55 (27.5)	40 (20.0)	
1-2 times/week	79 (39.5)	73 (36.5)	
3-6 times/week	8 (4.0)	22 (11.0)	
1 time/day	2 (1.0)	1 (0.5)	
>2 times/day	0	2 (1.0)	
Potato fried			0.001
Never/rarely	72 (36.0)	35 (17.5)	
1-3 times/month	34 (17.0)	52 (26.0)	
1-2 times/week	57 (28.5)	74 (37.0)	
3-6 times/week	8 (4.0)	11 (5.5)	
1 time/day	3 (1.5)	4 (2.0)	
>2 times/day	0	0	
Stuffed peppers, grape leaves, cabbage with rice			0.27
Never/rarely	62 (31.0)	74 (37.0)	
1-3 times/month	40 (20.0)	30 (15.0)	
1-2 times/week	68 (34.0)	72 (36.0)	
3-6 times/week	1 (0.5)	0	
1 time/day	3 (1.5)	0	
>2 times/day	0	0	
Stuffed peppers, grape leaves, cabbage with rice and meat			0.85
Never/rarely	103 (51.5)	101 (50.5)	
1-3 times/month	4 (2.0)	8 (4.0)	
1-2 times/week	66 (33.0)	65 (32.5)	
3-6 times/week	1 (0.5)	2 (1.0)	
1 time/day	0	0	
>2 times/day	0	0	
Vegetables cooked, including ladera foods			0.88
Never/rarely	14 (7.0)	14 (7.0)	
1-3 times/month	114 (57.0)	109 (54.5)	
1-2 times/week	21 (10.5)	33 (16.5)	
3-6 times/week	22 (11.0)	17 (8.5)	
1 time/day	3 (1.5)	2 (1.0)	
>2 times/day	0	1 (0.5)	
Vegetables pickled			0.65
Never/rarely	148 (74.0)	153 (76.5)	
1-3 times/month	5 (2.5)	4 (2.0)	
1-2 times/week	21 (10.5)	19 (9.5)	

3-6 times/week	0	0	
1 time/day	0	0	
>2 times/day	0	0	
Fruit canned			0.12
Never/rarely	148 (74.0)	158 (79.0)	
1-3 times/month	2 (1.0)	2 (1.0)	
1-2 times/week	21 (10.5)	15 (7.5)	
3-6 times/week	2 (1.0)	1 (0.5)	
1 time/day	1 (0.5)	0	
>2 times/day	0	0	
Fruit juice canned			0.92
Never/rarely	98 (49.0)	99 (49.5)	
1-3 times/month	28 (14.0)	30 (15.0)	
1-2 times/week	23 (11.5)	18 (9.0)	
3-6 times/week	17 (8.5)	21 (10.5)	
1 time/day	7 (3.5)	7 (3.5)	
>2 times/day	1 (0.5)	1 (0.5)	
Olives			0.006
Never/rarely	55 (27.5)	68 (34.0)	
1-3 times/month	32 (16.0)	35 (17.5)	
1-2 times/week	25 (12.5)	39 (19.5)	
3-6 times/week	24 (12.0)	14 (7.0)	
1 time/day	15 (7.5)	8 (4.0)	
>2 times/day	23 (11.5)	12 (6.0)	
Wine			0.36
Never/rarely	77 (38.5)	72 (36.0)	
1-3 times/month	42 (21.0)	42 (21.0)	
1-2 times/week	41 (20.5)	39 (19.5)	
3-6 times/week	8 (4.0)	18 (9.0)	
1 time/day	5 (2.5)	5 (2.5)	
>2 times/day	1 (0.5)	0	
Beer			0.14
Never/rarely	113 (56.5)	123 (61.5)	
1-3 times/month	17 (8.5)	22 (11.0)	
1-2 times/week	38 (19.0)	25 (12.5)	
3-6 times/week	4 (2.0)	6 (3.0)	
1 time/day	1 (0.5)	0	
>2 times/day	1 (0.5)	0	
Tahini			0.22
Never/rarely	107 (53.5)	132 (66.0)	
1-3 times/month	28 (14.0)	11 (5.5)	
1-2 times/week	27 (13.5)	21 (10.5)	
3-6 times/week	6 (3.0)	1 (0.5)	
1 time/day	5 (2.5)	10 (5.0)	
>2 times/day	1 (0.5)	1 (0.5)	

Table S5. Intake of ultra-processed foods based on food frequency questionnaire in fasters and non-fasters.

Variables	Fasters (n=174)	Non-fasters (n=176)	p-value
	n (%)	n (%)	
Sausage turkey			0.21
Never/rarely	71 (35.5)	62 (31.0)	
1-3 times/month	48 (24.0)	51 (25.5)	
1-2 times/week	26 (13.0)	26 (13.0)	
3-6 times/week	21 (10.5)	24 (12.0)	
1 time/day	6 (3.0)	11 (5.5)	
>2 times/day	2 (1.0)	2 (1.0)	
Sausage pork			0.003
Never/rarely	108 (54.0)	88 (44.0)	
1-3 times/month	28 (14.0)	27 (13.5)	
1-2 times/week	26 (13.0)	35 (17.5)	
3-6 times/week	10 (5.0)	19 (9.5)	
1 time/day	2 (1.0)	6 (3.0)	
>2 times/day	0	1 (0.5)	
Milk condensed full fat			0.79
Never/rarely	165 (82.5)	164 (82.0)	
1-3 times/month	1 (0.5)	0	
1-2 times/week	5 (2.5)	11 (5.5)	
3-6 times/week	1 (0.5)	1 (0.5)	
1 time/day	2 (1.0)	0	
>2 times/day	0	0	
Milk fresh full fat			0.61
Never/rarely	105 (52.5)	110 (55.0)	
1-3 times/month	14 (7.0)	8 (4.0)	
1-2 times/week	13 (6.5)	8 (4.0)	
3-6 times/week	26 (13.0)	28 (14.0)	
1 time/day	12 (6.0)	15 (7.5)	
>2 times/day	4 (2.0)	7 (3.5)	
Milk chocolate beverage non fat			0.22
Never/rarely	172 (86.0)	171 (85.5)	
1-3 times/month	0	0	
1-2 times/week	2 (1.0)	4 (2.0)	
3-6 times/week	0	1 (0.5)	
1 time/day	0	0	
>2 times/day	0	0	
Milk chocolate beverage full fat			0.28
Never/rarely	159 (79.5)	153 (76.5)	

1-3 times/month	1 (0.5)	3 (1.5)	
1-2 times/week	12 (6.0)	19 (9.5)	
3-6 times/week	2 (1.0)	1 (0.5)	
1 time/day	0	0	
>2 times/day	0	0	
Yogurt cow full fat			0.10
Never/rarely	119 (59.5)	108 (54.0)	
1-3 times/month	24 (12.0)	24 (12.0)	
1-2 times/week	21 (10.5)	30 (15.0)	
3-6 times/week	8 (4.0)	9 (4.5)	
1 time/day	1 (0.5)	4 (2.0)	
>2 times/day	1 (0.5)	1 (0.5)	
Yogurt dessert			0.91
Never/rarely	150 (75)	1 (0.5)	
1-3 times/month	7 (3.5)	152 (76.0)	
1-2 times/week	14 (7.0)	4 (2.0)	
3-6 times/week	3 (1.5)	15 (7.5)	
1 time/day	0	3 (1.5)	
>2 times/day	0	1 (0.5)	
Pudding milk or rice, full fat			0.78
Never/rarely	142 (71.0)	145 (72.5)	
1-3 times/month	4 (2.0)	5 (2.5)	
1-2 times/week	28 (14.0)	26 (13.0)	
3-6 times/week	0	0	
1 time/day	0	0	
>2 times/day	0	0	
Bagel and koulouri stuffed			0.42
Never/rarely	149 (74.5)	149 (74.5)	
1-3 times/month	9 (4.5)	3 (1.5)	
1-2 times/week	14 (7.0)	21 (10.5)	
3-6 times/week	2 (1.0)	3 (1.5)	
1 time/day	0	0	
>2 times/day	0	0	
Pita bread			0.15
Never/rarely	162 (81.0)	156 (78.0)	
1-3 times/month	2 (1.0)	6 (3.0)	
1-2 times/week	9 (4.5)	9 (4.5)	
3-6 times/week	1 (0.5)	5 (2.5)	
1 time/day	0	0	
>2 times/day	0	0	
Pita bread Cypriot			0.89
Never/rarely	156 (78.0)	158 (79.0)	
1-3 times/month	1 (0.5)	1 (0.5)	
1-2 times/week	17 (8.5)	16 (8.0)	
3-6 times/week	0	0	
1 time/day	0	1 (0.5)	

>2 times/day	0	0	
Pita bread round			0.52
Never/rarely	115 (57.5)	113 (56.5)	
1-3 times/month	16 (8.0)	16 (8.0)	
1-2 times/week	42 (21.0)	42 (21.0)	
3-6 times/week	1 (0.5)	5 (2.5)	
1 time/day	0	0	
>2 times/day	0	0	
Marmalade			0.25
Never/rarely	85 (42.5)	99 (49.5)	
1-3 times/month	41 (20.5)	32 (16.0)	
1-2 times/week	29 (14.5)	30 (15.0)	
3-6 times/week	9 (4.5)	9 (4.5)	
1 time/day	9 (4.5)	5 (2.5)	
>2 times/day	1 (0.5)	1 (0.5)	
Tarama dip			
Never/rarely	124 (62.0)	146 (73.0)	
1-3 times/month	14 (7.0)	9 (4.5)	
1-2 times/week	34 (17.0)	17 (8.5)	
3-6 times/week	1 (0.5)	2 (1.0)	
1 time/day	0	1 (0.5)	
>2 times/day	1 (0.5)	1 (0.5)	
Salad dips traditional, including tirokauteri, melitzanosalata			0.09
Never/rarely	92 (46.0)	80 (40.0)	
1-3 times/month	32 (16.0)	33 (16.5)	
1-2 times/week	42 (21.0)	51 (25.5)	
3-6 times/week	7 (3.5)	9 (4.5)	
1 time/day	1 (0.5)	2 (1.0)	
>2 times/day	0	1 (0.5)	
Ketchup			0.002
Never/rarely	119 (59.5)	98 (49.0)	
1-3 times/month	22 (11.0)	21 (10.5)	
1-2 times/week	31 (15.5)	47 (23.5)	
3-6 times/week	2 (1.0)	10 (5.0)	
1 time/day	0	0	
>2 times/day	0	0	
Mustard			0.014
Never/rarely	79 (39.5)	63 (31.5)	
1-3 times/month	30 (15.0)	32 (16.0)	
1-2 times/week	34 (17.0)	35 (17.5)	
3-6 times/week	13 (6.5)	11 (5.5)	
1 time/day	2 (1.0)	3 (1.5)	
>2 times/day	16 (8.0)	32 (16.0)	
Pizza			0.174
Never/rarely	76 (38.0)	64 (32.0)	

1-3 times/month	18 (9.0)	21 (10.5)	
1-2 times/week	77 (38.5)	86 (43.0)	
3-6 times/week	3 (1.5)	5 (2.5)	
1 time/day	0	0	
>2 times/day	0	0	
Lemonade, orangeade with sugar			0.17
Never/rarely	143 (71.5)	137 (68.5)	
1-3 times/month	11 (5.5)	11 (5.5)	
1-2 times/week	15 (7.5)	16 (8.0)	
3-6 times/week	2 (1.0)	8 (4.0)	
1 time/day	3 (1.5)	4 (2.0)	
>2 times/day	0	0	
Fizzy drink cola, light and zero			0.96
Never/rarely	154 (77.0)	156 (78.0)	
1-3 times/month	5 (2.5)	4 (2.0)	
1-2 times/week	13 (6.5)	14 (7.0)	
3-6 times/week	2 (1.0)	2 (1.0)	
1 time/day	0	0	
>2 times/day	0	0	
Fizzy drink other, light and zero			0.09
Never/rarely	166 (83.0)	163 (81.5)	
1-3 times/month	5 (2.5)	2 (1.0)	
1-2 times/week	3 (1.5)	11 (5.5)	
3-6 times/week	0	0	
1 time/day	0	0	
>2 times/day	0	0	
Fizzy drink cola, with sugar			0.52
Never/rarely	141 (70.5)	138 (69)	
1-3 times/month	12 (6.0)	16 (8)	
1-2 times/week	19 (9.5)	16 (8)	
3-6 times/week	1 (0.5)	5 (2.5)	
1 time/day	1 (0.5)	1 (0.5)	
>2 times/day	0	0	
Fizzy drink other, with sugar			0.09
Never/rarely	154 (77.0)	148 (74.0)	
1-3 times/month	7 (3.5)	8 (4.0)	
1-2 times/week	12 (6.0)	12 (6.0)	
3-6 times/week	1 (0.5)	8 (4.0)	
1 time/day	0	0	
>2 times/day	0	0	
Energy drinks			0.43
Never/rarely	0	0	
1-3 times/month	166 (83.0)	170 (85.0)	
1-2 times/week	2 (1.0)	2 (1.0)	
3-6 times/week	3 (1.5)	3 (1.5)	

1 time/day	3 (1.5)	1 (0.5)	
>2 times/day	0	0	
Isotonic drinks			0.99
Never/rarely	172 (86.0)	173 (86.5)	
1-3 times/month	0	2 (1.0)	
1-2 times/week	2 (1.0)	1 (0.5)	
3-6 times/week	0	0	
1 time/day	0	0	
>2 times/day	0	0	
Margarine			
Never/rarely	72 (36.0)	95 (47.5)	
1-3 times/month	41 (20.5)	37 (18.5)	
1-2 times/week	28 (14.0)	20 (10.0)	
3-6 times/week	19 (9.5)	12 (6.0)	
1 time/day	8 (4.0)	11 (5.5)	
>2 times/day	6 (3.0)	1 (0.5)	
Becel margarine			0.59
Never/rarely	142 (71.0)	142 (71.0)	
1-3 times/month	10 (5.0)	11 (5.5)	
1-2 times/week	12 (6.0)	8 (4.0)	
3-6 times/week	4 (2.0)	6 (3.0)	
1 time/day	2 (1.0)	4 (2.0)	
>2 times/day	4 (2.0)	5 (2.5)	
Mayonnaise full fat			0.69
Never/rarely	141 (70.5)	142 (71.0)	
1-3 times/month	12 (6.0)	11 (5.5)	
1-2 times/week	21 (10.5)	22 (11)	
3-6 times/week	0	0	
1 time/day	0	0	
>2 times/day	0	1 (0.5)	
Mayonnaise light			0.71
Never/rarely	159 (79.5)	166 (83.0)	
1-3 times/month	3 (1.5)	0	
1-2 times/week	8 (4.0)	6 (3.0)	
3-6 times/week	4 (2.0)	2 (1.0)	
1 time/day	0	1 (0.5)	
>2 times/day	0	1 (0.5)	
Donut, loukoumas			0.10
Never/rarely	152 (76.0)	145 (72.5)	
1-3 times/month	4 (2.0)	2 (1.0)	
1-2 times/week	18 (9.0)	28 (14.0)	
3-6 times/week	0	1 (0.5)	
1 time/day	0	0	
>2 times/day	0	0	
Dessert spoon, traditional			0.17
Never/rarely	86 (43.0)	106 (53.0)	

1-3 times/month	34 (17.0)	23 (11.5)	
1-2 times/week	42 (21.0)	38 (19.0)	
3-6 times/week	9 (4.5)	3 (1.5)	
1 time/day	3 (1.5)	6 (3.0)	
>2 times/day	0	0	
Desserts with chocolate			0.88
Never/rarely	65 (32.5)	72 (36.0)	
1-3 times/month	44 (22.0)	36 (18.0)	
1-2 times/week	47 (23.5)	51 (25.5)	
3-6 times/week	11 (5.5)	8 (4.0)	
1 time/day	5 (2.5)	7 (3.5)	
>2 times/day	2 (1.0)	2 (1.0)	
Biscuits cake plain			0.68
Never/rarely	53 (26.5)	59 (29.5)	
1-3 times/month	53 (26.5)	51 (25.5)	
1-2 times/week	36 (18.0)	39 (19.5)	
3-6 times/week	19 (9.5)	15 (7.5)	
1 time/day	13 (6.5)	7 (3.5)	
>2 times/day	0	5 (2.5)	
Biscuits cake with chocolate			0.12
Never/rarely	87 (43.5)	103 (51.5)	
1-3 times/month	29 (14.5)	30 (15.0)	
1-2 times/week	43 (21.5)	30 (15.0)	
3-6 times/week	10 (5.0)	8 (4.0)	
1 time/day	5 (2.5)	4 (2.0)	
>2 times/day	0	1 (0.5)	
Nougat			0.09
Never/rarely	113 (56.5)	127 (63.5)	
1-3 times/month	12 (6.0)	14 (7.0)	
1-2 times/week	46 (23.0)	32 (16.0)	
3-6 times/week	2 (1.0)	3 (1.5)	
1 time/day	1 (0.5)	0	
>2 times/day	0	0	
Nougat with nuts			0.15
Never/rarely	131 (65.5)	145 (72.5)	
1-3 times/month	12 (6.0)	6 (3.0)	
1-2 times/week	27 (13.5)	22 (11.0)	
3-6 times/week	2 (1.0)	3 (1.5)	
1 time/day	2 (1.0)	0	
>2 times/day	0	0	
Halvah tahini			0.24
Never/rarely	106 (53.0)	118 (59.0)	
1-3 times/month	10 (5.0)	10 (5.0)	
1-2 times/week	52 (26.0)	43 (21.5)	
3-6 times/week	3 (1.5)	3 (1.5)	
1 time/day	2 (1.0)	1 (0.5)	

>2 times/day	1 (0.5)	1 (0.5)	
Halvah semolina			0.33
Never/rarely	134 (67.0)	141 (70.5)	
1-3 times/month	3 (1.5)	2 (1.0)	
1-2 times/week	33 (16.5)	32 (16)	
3-6 times/week	2 (1.0)	1 (0.5)	
1 time/day	1 (0.5)	0	
>2 times/day	1 (0.5)	0	
Cacao powder or beverages			0.18
Never/rarely	141 (70.5)	131 (65.5)	
1-3 times/month	10 (5.0)	11 (5.5)	
1-2 times/week	14 (7.0)	23 (11.5)	
3-6 times/week	4 (2.0)	7 (3.5)	
1 time/day	5 (2.5)	2 (1.0)	
>2 times/day	0	2 (1.0)	
Chocolate powder or beverages			0.84
Never/rarely	140 (70.0)	142 (71)	
1-3 times/month	8 (4.0)	11 (5.5)	
1-2 times/week	25 (12.5)	22 (11.0)	
3-6 times/week	1 (0.5)	0	
1 time/day	0	1 (0.5)	
>2 times/day	0	0	
Ouzo, raki, and tsipouro			0.06
Never/rarely	125 (62.5)	113 (56.5)	
1-3 times/month	17 (8.5)	16 (8.0)	
1-2 times/week	26 (13)	39 (19.5)	
3-6 times/week	3 (1.5)	1 (0.5)	
1 time/day	3 (1.5)	6 (3.0)	
>2 times/day	0	1 (0.5)	
Spirit beverages			0.05
Never/rarely	151 (75.5)	141 (70.5)	
1-3 times/month	6 (3.0)	9 (4.5)	
1-2 times/week	17 (8.5)	21 (10.5)	
3-6 times/week	0	5 (2.5)	
1 time/day	0	0	
>2 times/day	0	0	
Corn flakes white			0.44
Never/rarely	140 (70.0)	139 (69.5)	
1-3 times/month	12 (6.0)	10 (5.0)	
1-2 times/week	10 (5.0)	9 (4.5)	
3-6 times/week	7 (3.5)	10 (5.0)	
1 time/day	4 (2.0)	8 (4.0)	
>2 times/day	1 (0.5)	0	
Corn flakes whole grain			0.77
Never/rarely	123 (61.5)	133 (66.5)	

1-3 times/month	16 (8.0)	13 (6.5)	
1-2 times/week	22 (11.0)	12 (6.0)	
3-6 times/week	7 (3.5)	10 (5.0)	
1 time/day	5 (2.5)	6 (3.0)	
>2 times/day	1 (0.5)	2 (1.0)	
Corn flakes with chocolate			0.58
Never/rarely	154 (77.0)	158 (79.0)	
1-3 times/month	5 (2.5)	1 (0.5)	
1-2 times/week	12 (6.0)	8 (4.0)	
3-6 times/week	3 (1.5)	7 (3.5)	
1 time/day	0	2 (1.0)	
>2 times/day	0	0	
Coco pops cereals			0.79
Never/rarely	159 (79.5)	163 (81.5)	
1-3 times/month	7 (3.5)	3 (1.5)	
1-2 times/week	6 (3.0)	5 (2.5)	
3-6 times/week	2 (1.0)	5 (2.5)	
1 time/day	0	0	
>2 times/day	0	0	
Muesli oats			0.88
Never/rarely	135 (67.5)	143 (71.5)	
1-3 times/month	15 (7.5)	5 (2.5)	
1-2 times/week	6 (3.0)	10 (5.0)	
3-6 times/week	12 (6.0)	11 (5.5)	
1 time/day	4 (2.0)	6 (3.0)	
>2 times/day	2 (1.0)	1 (0.5)	
Cereal bars			0.44
Never/rarely	136 (68.0)	133 (66.5)	
1-3 times/month	16 (8.0)	14 (7.0)	
1-2 times/week	15 (7.5)	20 (10.0)	
3-6 times/week	4 (2.0)	7 (3.5)	
1 time/day	3 (1.5)	1 (0.5)	
>2 times/day	0	1 (0.5)	
Peanut butter			0.72
Never/rarely	161 (80.5)	166 (83.0)	
1-3 times/month	5 (2.5)	0	
1-2 times/week	7 (3.5)	10 (5.0)	
3-6 times/week	0	0	
1 time/day	1 (0.5)	0	
>2 times/day	0	0	
Ice cream			0.37
Never/rarely	133 (66.5)	143 (71.5)	
1-3 times/month	11 (5.5)	6 (3.0)	
1-2 times/week	28 (14.0)	26 (13.0)	
3-6 times/week	2 (1.0)	1 (0.5)	
1 time/day	0	0	

>2 times/day	0	0	
Potato chips			0.29
Never/rarely	117 (58.5)	125 (62.5)	
1-3 times/month	13 (6.5)	16 (8.0)	
1-2 times/week	42 (21.0)	33 (16.5)	
3-6 times/week	1 (0.5)	2 (1.0)	
1 time/day	0	0	
>2 times/day	0	0	

Table S6. Logistic regression analysis for UPF food items

Variables in the Equation

		B	S.E.	Wald	df	Sig.	Exp(B)	95% C.I.for EXP(B)	
								Lower	Upper
Step 1[a]	bagel_koulouri_stuffed	-.044	.280	.024	1	.876	.957	.553	1.657
	bars	-.204	.232	.775	1	.379	.815	.517	1.285
	biscuits_cake_plain	-.007	.147	.002	1	.962	.993	.744	1.325
	biscuits_cake_w_chocolate	-.276	.186	2.211	1	.137	.758	.527	1.092
	sausage_pork	.044	.165	.070	1	.792	1.045	.756	1.444
	sausage_turkey	.055	.142	.148	1	.700	1.056	.800	1.394
	milk_condensed_full_fat	-.529	.489	1.170	1	.279	.589	.226	1.537
	milk_fresh_full_fat_3.5	.041	.108	.144	1	.704	1.042	.843	1.289
	milk_choco_beverage_wholemilk	.195	.336	.335	1	.562	1.215	.629	2.348
	yogurt_cow_full_fat_10	-.224	.168	1.785	1	.182	.799	.575	1.110
	yogurt_dessert	-.013	.262	.002	1	.962	.988	.591	1.650
	pita_bread	-.090	.334	.072	1	.788	.914	.475	1.758
	pita_bread_round	-.236	.213	1.224	1	.269	.790	.521	1.199
	pizza	-.012	.178	.004	1	.947	.988	.697	1.401
	fizzy_drink_w_sugar_other	-.392	.364	1.160	1	.282	.676	.331	1.379
	fizzy_drink_cola_w_sugar	-.268	.294	.833	1	.361	.765	.430	1.360
	fiizy_drink_light_other	.074	.531	.019	1	.889	1.077	.380	3.047
	energy_drinks	-.254	.705	.130	1	.719	.776	.195	3.089
	isotonic_drinks	.724	1.059	.468	1	.494	2.063	.259	16.426
	becel_1teaspoon	.100	.141	.496	1	.481	1.105	.837	1.458
	margarine_1teaspoon	.125	.130	.934	1	.334	1.133	.879	1.461

mayonnaise_1tablespoon	-.153	.245	.390	1	.532	.858	.531	1.386
mayonnaise_light_1tablespoon	.065	.307	.044	1	.833	1.067	.584	1.949
dessert_homemade_koutaliou	.113	.159	.508	1	.476	1.120	.820	1.530
dessert_w_chocolate_pasta	-.133	.149	.790	1	.374	.876	.653	1.174
donut_loukoumas	-.237	.282	.706	1	.401	.789	.454	1.371
nougat	.286	.207	1.901	1	.168	1.331	.886	1.999
nougat_w_nuts	-.285	.241	1.394	1	.238	.752	.469	1.207
halvah_tahini	-.140	.175	.642	1	.423	.869	.617	1.224
halvah_semolina	.250	.216	1.346	1	.246	1.284	.842	1.960
cacao_powder	-.021	.167	.015	1	.902	.980	.706	1.359
chocolate_powder_or_beverage	-.350	.272	1.654	1	.198	.705	.414	1.201
ouzo_raki_tsipouro	.236	.165	2.053	1	.152	1.266	.917	1.750
spirit_beverages	-.330	.277	1.422	1	.233	.719	.418	1.237
corn_flakes_white	.004	.159	.000	1	.982	1.004	.735	1.371
corn_flakes_wholemeal	-.104	.177	.341	1	.559	.902	.637	1.276
corn_flakes_w_chocolate	-.122	.273	.201	1	.654	.885	.518	1.511
muesli_oats	-.081	.155	.272	1	.602	.922	.681	1.249
peanut_butter	-.041	.361	.013	1	.909	.960	.473	1.948
ice_cream	-.223	.237	.887	1	.346	.800	.503	1.272
chips_potato	-.173	.230	.562	1	.453	.841	.536	1.321
salad_dip_tirokauteri_melitzanosalata	-.006	.167	.001	1	.972	.994	.716	1.380
ketchup_1tablespoon	.327	.203	2.595	1	.107	1.387	.932	2.066
mustard_1tablespoon	.042	.119	.127	1	.722	1.043	.827	1.316
Constant	9.336	6830.394	.000	1	.999	11336.828		

a. Variable(s) entered on step 1: bagel_koulouri_stuffed, bars, biscuits_cake_plain, biscuits_cake_w_chocolate, sausage_pork, sausage_turkey, milk_condensed_full_fat, milk_fresh_full_fat_3.5, milk_choco_beverage_wholemilk, yogurt_cow_full_fat_10, yogurt_dessert, pita_bread, pita_bread_round, pizza, fizzy_drink_w_sugar_other, fizzy_drink_cola_w_sugar, fiizy_drink_light_other, energy_drinks, isotonic_drinks, becel_1teaspoon, margarine_1teaspoon, mayonnaise_1tablespoon, mayonnaise_light_1tablespoon, dessert_homemade_koutaliou, dessert_w_chocolate_pasta, donut_loukoumas, nougat, nougat_w_nuts, halvah_tahini, halvah_semolina, cacao_powder, chocolate_powder_or_beverage, ouzo_raki_tsipouro, spirit_beverages, corn_flakes_white, corn_flakes_wholemeal, corn_flakes_w_chocolate, muesli_oats, peanut_butter, ice_cream, chips_potato, salad_dip_tirokauteri_melitzanosalata, ketchup_1tablespoon, mustard_1tablespoon.
